# Clinical implementation of RTT-only CBCT-guided online adaptive focal radiotherapy for bladder cancer

**DOI:** 10.1016/j.ctro.2024.100884

**Published:** 2024-11-02

**Authors:** K. Goudschaal, S. Azzarouali, J. Visser, M. Admiraal, J. Wiersma, N. van Wieringen, A. de la Fuente, M. Piet, L. Daniels, D. den Boer, M. Hulshof, A. Bel

**Affiliations:** a Amsterdam UMC Location University of Amsterdam, Radiation Oncology, Meibergdreef 9, Amsterdam, the Netherlands; b Amsterdam UMC Location Vrije Universiteit Amsterdam, Radiation Oncology, De Boelelaan 1117, Amsterdam, the Netherlands; c Cancer Center Amsterdam, Cancer Therapy, Treatment and Quality of Life, Amsterdam, the Netherlands; d The Netherlands Cancer Institute, Radiation Oncology, the Netherlands

**Keywords:** Online adaptive focal radiotherapy, Bladder cancer, CBCT-guided RTT-only workflow, Bladder sparing treatment, RTT-only

## Abstract

**Purpose:**

The study assesses the clinical implementation of radiation therapist (RTT)-only Conebeam CT (CBCT)-guided online adaptive focal radiotherapy (oART) for bladder cancer, by describing the training program, analyzing the workflow and monitoring patient experience.

**Materials and methods:**

Bladder cancer patients underwent treatment (20 sessions) on a ring-based linac (Ethos, Varian, a Siemens Healthineers Company, USA). Commencing April 2021, 14 patients were treated by RTTs supervised by the Radiation Oncologist (RO) and Medical Physics Expert (MPE) in a multidisciplinary workflow. From March 2022, 14 patients were treated solely by RTTs. RTT training included target delineation lessons and practicing oART in a simulation environment. We analyzed the efficiency of the RTT-only workflow regarding session time, adjustments by RTTs, attendance of the RO and MPE at the linac, and qualitative assessment of gross tumor volume (GTV) delineation. Patient experience was monitored through questionnaires.

**Results:**

A training program resulted in a skilled team of RTTs, ROs and MPEs.

The RTT-only workflow demonstrated shorter session times compared to the multidisciplinary approach. Among 14 patients treated using the RTT-only workflow, RTTs adjusted 99% of bladder volumes and 44% of GTV. 79% of the sessions proceeded without MPEs and ROs. All GTV delineations were RO-approved, thus considered clinically acceptable, and 87% required minor or no adjustments. Patient satisfaction was reported in 18 of 21 cases.

**Conclusions:**

The RTT-only oART workflow for bladder cancer, complemented by a training program and on-call support from ROs and MPEs, demonstrated success. Patient experience is positive. It is currently introduced as standard in our clinic.

## Highlights

• RTT-only workflow is feasible for bladder cancer online focal adaptive radiotherapy.
• A competent multidisciplinary team is key for implementing RTT-only workflow.
• RTT-only workflow reduces clinicians’ workload.
• Improves efficiency at the linac.
• RTT-only workflow is introduced as standard with positive experiences for patients.

## Graphical abstract

**Figure:**
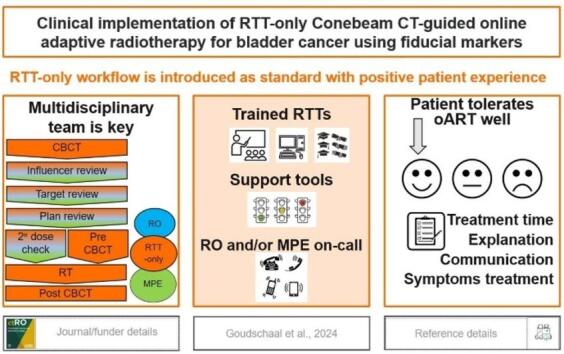

## 1 Introduction

Bladder cancer ranks among the top ten most common cancers for both men and women, with its incidence increasing steadily with age [1]. Approximately 20–30 % of bladder cancer patients have muscle-invasive bladder cancer (MIBC) [2]. Treatment options include radical cystectomy or trimodality treatment [2], which involves *trans*-urethral resection of the bladder tumor followed by chemoradiotherapy. Trimodality therapy is a good option for patients who are either ineligible for radical cystectomy or choose not to undergo it [2], [3], [4], [5], [6], [7], [8], [9], [10], as it preserves sexual and urinary functions [11].

Treating MIBC patients with radiotherapy is challenging due to highly variable bladder shape, size and filling, as well as poor tumor visualization on Computed Tomography (CT) and Cone-beam CT (CBCT) [12], [13], [14]. Tumor visualization is especially important in the case of focal RT to ensure that the high radiation dose remains focused on the tumor bed or residual tumor. There were online adaptive studies performed using focal RT [15], [16] and Library of Plans (LoP) [17], [18]. To account for the interfraction variation in bladder filling, several options are available: LoP [12], [19], [20], [21], [22]; Magnetic Resonance (MR)-guided online adaptive radiotherapy (oART) [13], [23]; and CBCT-guided oART [24], [25]. Despite drinking and voiding instructions, bladder volume variation can still be significant [26], [27], potentially leading to over- or under-treatment [28].

LoP has on-couch times averaging 9–19 min [29], [30], [31], oART has longer on-couch times than LoP, ranging from 30 to 40 min for MR-guided oART [13], [23], [32], [33], [34], [35], [36] and 14–29 min for CBCT-guided oART [24], [37], [38], [39], [40], [41], [42], [43]. Both LoP and oART are more labor-intensive than regular radiotherapy treatments, with LoP requiring more time during the pretreatment phase for the creation of the library, and oART requiring more time during treatment sessions due to daily plan generation. Bladder filling over time affects oART workflows by influencing the time and accuracy of adaptive plan generation, leading to a larger PTV margin. Therefore we take in account imaging, treatment time, and drinking instructions. For oART, balancing the time needed for daily plan generation with the time it takes for the bladder to fill can be challenging [12]. These modalities typically increase the workload, particularly when a multidisciplinary team is present for each session.

As oART evolves, Radiation Therapists (RTTs) are taking on more redefined central roles, with a broader shift from conventional roles at the linac [34] towards RTT-only workflows becoming evident [44], [45], [46]. With this shift currently underway, there are some initial examples of RTTs leading the oART workflow under the supervision of the Radiation Oncologist (RO) and the Medical Physics Expert (MPE) [38] or with the help of decision support tools and RO and MPE on-call [42], [44]. Action protocols [47], [48] help RTTs address anatomical changes on CBCT, which is crucial for RTT-only oART [38]. Training RTTs for tasks like target delineation is essential [37], [38], [39], with studies showing their accuracy can match that of ROs [32], [49]. These examples demonstrate the expanding and essential role of RTTs in modern radiotherapy, leading to improved efficiency and more patient-centered care.

The trend towards RTT-only oART requires ongoing skill and competence development [44], [50], during-accrual image guided radiation therapy (IGRT) quality assurance (QA) [51], as well as better coordination of RO and MPE availability, which is essential for widespread implementation. CBCT-guided RTT-only approaches were successfully implemented for rectal and prostate cancer [38], [39]. Åström et al. reported a similar RTT-led workflow to the one we use for non-focal whole bladder cancer oART [52]. However, a comprehensive analysis of the implementation and RTT-only workflow for focal bladder cancer oART has not yet been reported, to the best of our knowledge. This study aimed to assess our clinical implementation of RTT-only CBCT-guided oART for focal bladder cancer, including nodal treatments, by describing the training program, analyzing the main workflow steps and monitoring patient experience.

## 2 Materials and methods

### 2.1 Study population

In a one year training stage, starting April 2021, “the multidisciplinary workflow”, 14 patients with MIBC underwent curative radiotherapy on a ring-based linac integrated with a CBCT and software platform for treatment planning and delivery (Ethos Therapy™, version 1.1, Varian a Siemens Healthineers Company, USA). Exclusion criteria for oART included bilateral hip prosthesis, height over 1.95 m, or inability to lie still for 20 min without voiding.

**Fig. 1:**
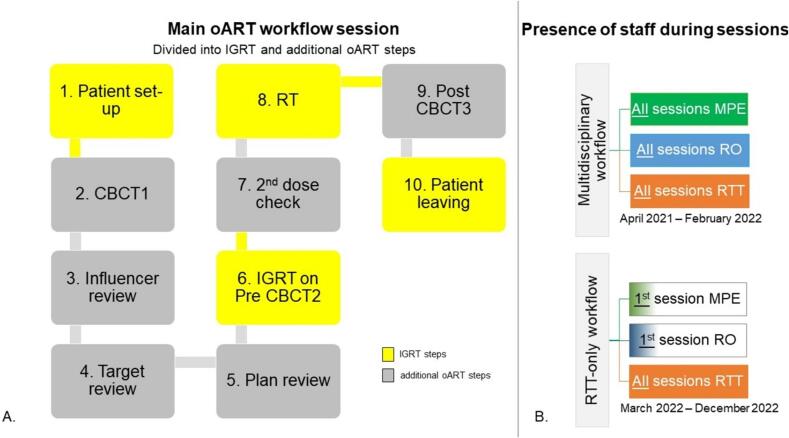
A) Flowchart of the main oART workflow session, divided into IGRT (yellow) and additional oART steps (grey). B) Presence of staff during oART sessions. Multidisciplinary workflow: Multidisciplinary involvement of the MPE and RO is required during all sessions. RTT-only workflow: The MPE and RO remained present for each patient’s first session. Abbreviations: oART (Online adaptive radiotherapy treatment planning), IGRT (image guided radiation therapy), MPE (Medical Physics Expert), RO (Radiation Oncologist) RTT (Radiation therapist). (For interpretation of the references to colour in this figure legend, the reader is referred to the web version of this article.)

After the multidisciplinary workflow, 14 patients were treated in a subsequent stage, “the RTT-only workflow”, details below (Fig. 1B).

Patients received radiotherapy with either a simultaneous integrated boost (SIB, 20 sessions of 2 Gy to the full bladder, urethra and pelvic lymph nodes – including the internal iliac, obturator, hypogastric and perivesical nodes up to the lower part of the sacroiliac joint –, combined with 2.75 Gy on the tumor bed or any residual tumor) or a sequential boost (5 sessions of 3 Gy with a full bladder on the tumor bed or any residual tumor, followed by 15 sessions of 2.67 Gy on the empty bladder, urethra and pelvic lymph nodes), combined with chemotherapy (Mitomycin-C/Capecitabine).

Patient characteristics, pretreatment information, and details from our previous study by Azzarouali et al. are summarized in Supplementary data 1.

In line with the regulations of the local ethics committee, 21 patients provided informed consent to participate in a study evaluating the feasibility and patient experience of oART. This evaluation was conducted during both the multidisciplinary and RTT-only workflows using questionnaires administered during treatment. An METC-waiver was granted on October 21th, 2021, by Amsterdam UMC, location AMC, under CMG number 111467.

### 2.2 Training program

An infrastructure for oART training and support was developed (Table A.3), this framework was also used in other oART target areas. RTTs received bladder target definition training from an RO and in-house training with feedback on gross tumor volume (GTV), clinical target volume (CTV) and organ-at-risk (OAR) delineation. They practiced oART in the online Ethos test environment (Emulator, version 1.1, Varian a Siemens Healthineers company, USA) under RO and MPE supervision, supported by a traffic light protocol [47], [48] (Fig. A.1).

RTTs were examined by specialist RTTs in the Emulator on their ability to independently execute the oART workflow and discuss its steps and consequences. After training, RTTs completed 10 clinical training sessions supervised by a trained RTT. The aim was for RTTs to operate independently, with ROs and MPEs on-call for support, in an RTT-only workflow.

ROs and MPEs were also trained on the software and the entire oART workflow to support RTTs remotely. Periodic case meetings were held to secure the RTT-only workflow and exchange multidisciplinary knowledge.

We evaluated the training program’s effectiveness by gathering structured feedback from trainers and assessing RTT performance during the first ten clinical training sessions under supervision of a trained RTT. The assessment aimed to determine whether RTTs could independently operate the oART workflow, in the context of having access to RO and MPE support for complex decision-making, if needed.

### 2.3 Pretreatment bladder oART

Fiducial markers (BioXmark, Nanovi A/S, Denmark) are cystoscopically placed1-3 days before the planning CT (pCT) by the urologist at the borders of the tumor bed or residual tumor. These markers may be used to counteract poor visibility of the tumor bed or residual tumor on pretreatment pCT and CBCT images without causing additional artefacts [25]. They assist in GTV localization and delineation for both the RO during pretreatment on pCT and the RTT during online sessions on CBCT [53], [54]. This enables a 20-minute CBCT-guided oART session time, with the goal of further reducing toxicity [3], [25], [30], [53], [54], [55].

Patients were instructed to drink 0.3L of water after voiding for a full bladder and avoid drinking 1.5 h before CT and oART. For an empty bladder, they were asked to empty their bladder 1.5 h before oART and refrain from drinking. The pCT was made in supine patient position with the arms on the chest and a knee support [25]. The RO delineated the GTV (tumor bed or any residual tumor) and CTV with the aid of fiducial markers on the pCT (Aria, Varian a Siemens Healthineers Company) using European Association of Urology (EAU) guidelines [56]. RTTs were responsible for delineating the OARs. Due to its importance in toxicity for this type of cancer patients, linked to its sometimes more difficult visibility, the small bowel was initially outlined on the pCT by the RO. This was only done when the small bowel was either inside or close to the planning target volume of the boost (PTV_boost_) and therefore at risk of receiving a high dose. The structures were subsequently adjusted by the RTT during online oART sessions within a 2 cm ring around the PTV_boost_ to minimize the maximum dose to this structure.

The reference treatment plan was generated with the Ethos treatment planning system (TPS) [25]. Further information on bladder cancer oART using SIB and fiducial markers can be found in our earlier study [25].

### 2.4 Online adaptive workflow

An initial CBCT (CBCT1) was acquired during the online adaptive workflow (Fig. 1A). The subsequent steps include influencer structure review, target review, and plan review [33], displaying the reference/pretreatment and adapted plan (see also [25], [43]), followed by a pre oART CBCT (CBCT2) and post oART CBCT (CBCT3). During the influencer structure review, the influencer structures (system-generated delineations using Artificial Intelligence) are generated and reviewed. These structures guide the deformation of target volumes as defined on the pCT to the daily anatomy through structure-guided deformable registration. Session time is defined as the time between the patient entering and leaving the treatment room. The on-couch time is defined as the time from the first image acquisition (CBCT1) until the end of RT, excluding the time spent on patient setup and post image acquisition (CBCT3).

#### 2.4.1 Multidisciplinary workflow: the first-year training stage

From April 2021, 14 patients were treated using the multidisciplinary workflow. RTTs, the RO and MPE were present at the linac for all treatment sessions (Fig. 1B).

#### 2.4.2 RTT-only workflow

In the RTT-only workflow, from March to December 2022, 14 patients were treated. Data on the main workflow steps were analyzed, followed by offline QA by the MPE, RTT and RO. Two trained RTTs executed the RTT-only workflow, with the MPE and RO present only during the first session and available on-call thereafter (Fig. 1B). The traffic light protocol was used as a support tool (see Fig. A.1 and Table A.4 for traffic light criteria). The oncology information system (OIS) facilitated multidisciplinary communication. Specialist RTTs informed the RO and/or MPE of any deviations. For each patient, a brief meeting was scheduled with the RO and MPE before the first treatment.

We analyzed the efficiency and efficacy of the online adaptive workflow for the multidisciplinary and RTT-only workflows, focusing on:

a. Comparing **session times** (patient entering and leaving the room; Fig. 1A) between the RTT-only and multidisciplinary workflows using a Mann-Whitney test (IBM SPSS Statistics for Windows, Version 28.0 (NY: IBM Corp).
b. Registering the number of sessions with **adjustments of Influencers, Targets and OAR** by RTT during the RTT-only stage (Fig. 1A, steps 3 and 4).
c. Recording **additional support** (RO and MPE) during RTT-only workflow.
d. A retrospective assessment was conducted on the **GTV delineation by RTTs**, as this was a new skill for them, using CBCT1 from sessions 1,3,5,10,15 and 20 within the RTT-only workflow. The delineations were scored by the RO in four categories: similar to RTT delineation; clinically acceptable, although minor adjustments <3 mm would have been made; major adjustments (>3mm) but within PTV_boost_, and unacceptable (outside PTV_boost_).
e. Evaluating OIS **feedback on offline QA**. RO performed offline target coverage checks on the CBCT2 for sessions 1–5 during the RTT-only workflow (Fig. 1A, step 6). The MPE conducted a retrospective random plan review on hot and cold spots, dose conformity, and deviations in monitor units (MUs) between the reference/pretreatment and adapted plans (Fig. 1A, step 5). Written feedback was provided in the OIS for deviations in target coverage and dosimetry according to the traffic light protocol.

### 2.5 Monitoring patient experience

From January 2022 to March 2023, patients completed in-house questionnaires (see Supplementary data 6) about their experience with the treatment duration during multidisciplinary and RTT-only workflows, including their ability to remain in the treatment position. The questionnaires also assessed discomfort due to bladder filling and any trends in discomfort throughout the treatment, possibly from radiation-induced complaints.

The discomfort questionnaires included one baseline questionnaire before oART and four follow-up questionnaires during session 5,10,15 and 20, all using a 5-point Likert scale. Additionally, a questionnaire measuring satisfaction with the treatment on Ethos used a 4-point Likert scale [57].

## 3 Results

### 3.1 Training program

The multidisciplinary training program resulted in a skilled team of 18 RTTs working independently, with 6 ROs and 7 MPEs on-call for support in an RTT-only workflow, and was thus successful.

### 3.2 Session time

**Fig. 2:**
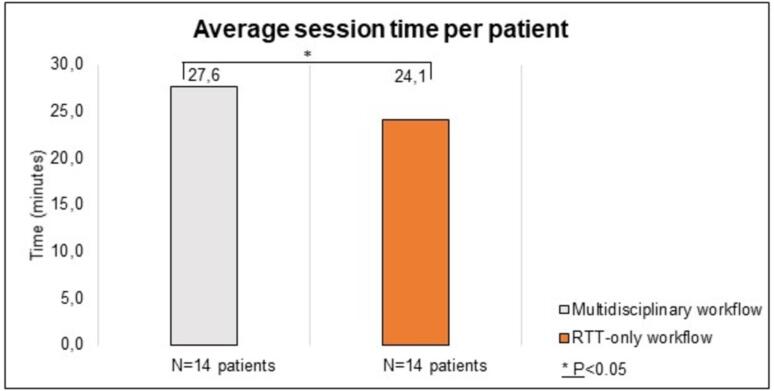
Average session time in minutes per patient for the multidisciplinary and RTT-only workflows. A total of 28 patients, 560 sessions, were treated with oART using a focal boost.

The session times of 28 patients (20 SIB, 8 sequential boost) (560 sessions) were recorded, with 14 patients treated in the multidisciplinary workflow and 14 in the RTT-only workflow. Comparing the average session times between the multidisciplinary and RTT-only workflows for both groups of 14 patients (Fig. 2) revealed that during RTT-only sessions, the overall session time (24.1 min, range 18–29 min) was significantly shorter (p < 0.05) compared to the multidisciplinary workflow (27.6 min, range 18–41 min).

### 3.3 Adjustments of influencers, targets and OAR

**Fig. 3:**
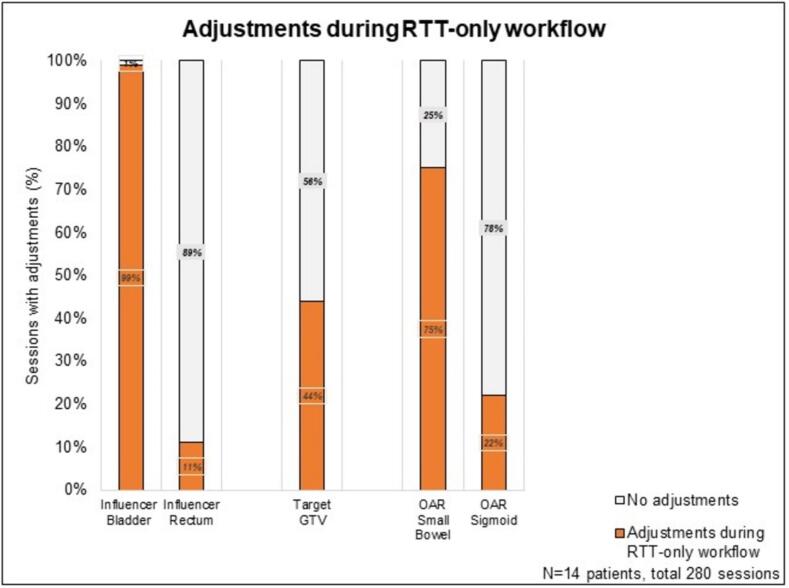
Adjustments made by RTTs to the automatically generated contours during the RTT-only workflow.

On the pretreatment imaging, the RO delineated the bladder and the GTV in 14 patients, while in 11 of them, the small bowel, located in or close to the PTV_boost_, was also delineated by the RO. Rectum and sigmoid were delineated by the RTT. In the RTT-only oART workflow, bladder adjustments due to interfraction bladder filling were made in nearly all sessions, rectum adjustments in 11 % of sessions, and GTVs were adjusted in 44 % of sessions. More adjustments were required for the small bowel compared to the sigmoid (Fig. 3).

### 3.4 Additional support during RTT-only workflow

**Fig. 4:**
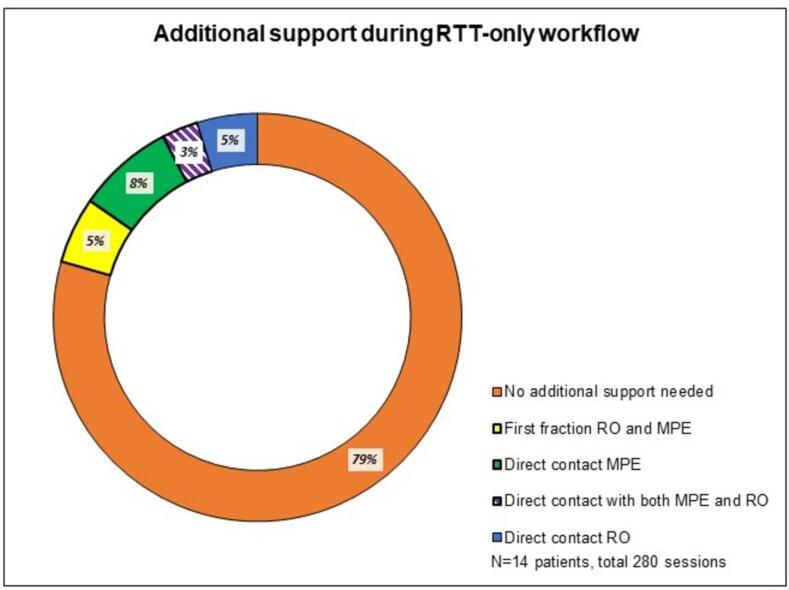
Additional support during RTT-only workflow. See Fig. A.2 for the number and the reasons of contact during the different steps of the RTT-only workflow.

Two trained RTTs conducted the RTT-only workflow, with the MPE and RO present only during each patient’s first session (14 times) and called upon if required during session 2–20 (46 times: RO directly 14 times, MPE directly 23 times, and additionally both RO and MPE simultaneously 9 times) (Fig. 4). The majority of treatments (220/280) were performed solely by RTTs. Direct contact with the RO (14 times) mainly occurred for questions about OAR review (specifically small bowel delineation), while contact with the MPE (23 times) was primarily regarding differences in MUs between the adapted plan and the reference/pretreatment plan. Both RO and MPE were contacted together (9 times) due to hotspots within PTV, OAR constraints (small bowel), and when the GTV was just inside PTV.

### 3.5 Assessment of GTV delineation

**Fig. 5:**
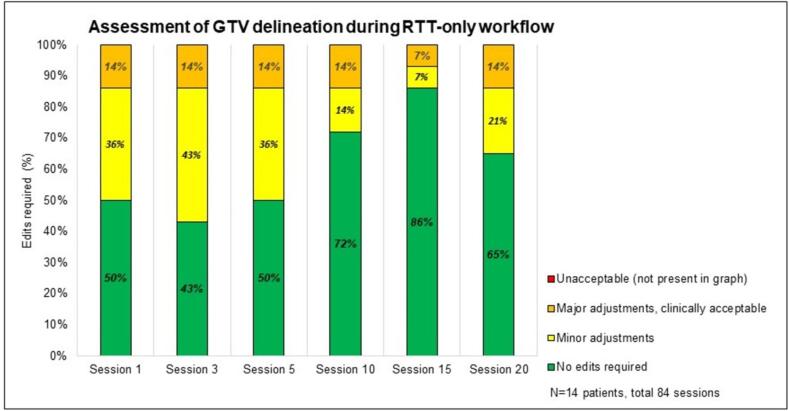
Qualitative assessment of GTV delineation on CBCT1 within the RTT-only workflow by the RO, retrospectively.

Eighty-four sessions (session 1,3,5,10,15 and 20 of 14 patients treated within the RTT-only workflow) had GTV delineations scored retrospectively in four categories by the RO (Fig. 5). In 51 sessions, the GTV delineation matched the RTT delineation. In 22 sessions, the GTV delineation was clinically acceptable, although adjustments within 3 mm would have been made. In 11 sessions, major adjustments (3 mm to 6 mm) would have been required, but the GTV is expected to be encompassed by the PTV_boost_, thus clinically acceptable. None of the assessed GTV delineations were unacceptable, defined as GTVs outside the PTV_boost_.

Analysis per patient showed that 9 out of 14 had no edits or minor adjustments required and five out of 14 patients scored orange. This means that major adjustments (3 mm to 6 mm) would have been required but the GTV was expected to be encompassed by the PTV_boost_ (Fig. A.3).

### 3.6 Feedback on offline QA to the RTT in the OIS

**Fig. 6:**
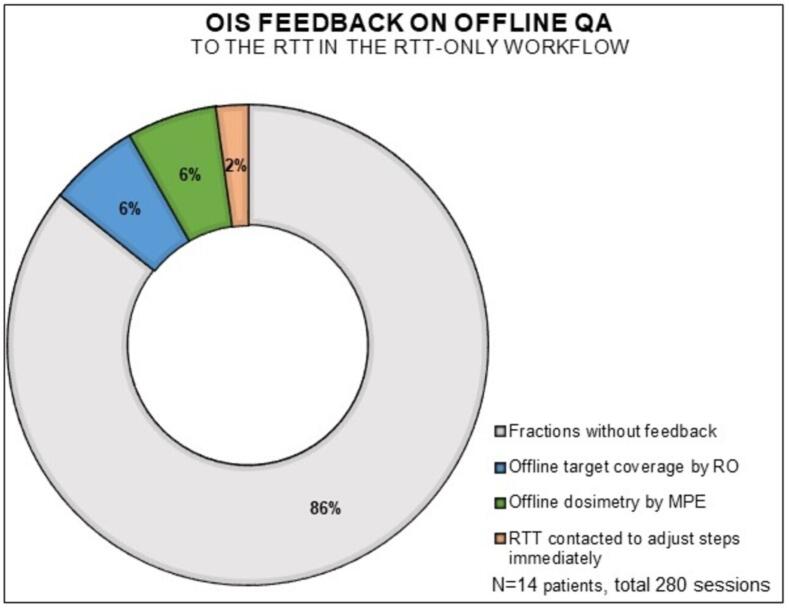
OIS feedback on offline QA fed back to the RTT.

The OIS feedback on offline QA to the RTT by the RO and MPE was analyzed. Most treatments (240/280) did not require feedback (Fig. 6). In 17/280 sessions, the MPE provided feedback to the RTT in the OIS about larger MUs deviations between the adapted and reference/pretreatment plan than previously allowed within the traffic light protocol (Fig. A.1). Similarly, in 17/280 sessions, the RO provided feedback to the RTT regarding adjustments to PTV margins due to intrafraction bladder filling, as outlined in the traffic light protocol. Additionally, the RO provided feedback about OAR adjustments, like not meeting small bowel constraints and coldspots due to the small bowel’s position in or close to the target area. If necessary, the RTT was contacted to immediately adjust the oART workflow (6/280) regarding the small bowel delineation and prioritizing GTV position verification of the GTV over the elective region.

### 3.7 Questionnaires

We recruited 21 bladder cancer patients undergoing treatment with oART on Ethos, encompassing both multidisciplinary and RTT-only workflows, to complete six in-house questionnaires: one on treatment satisfaction, one on basic complaints, and four on follow-up complaints. Three patients dropped out due to health changes. On average, each patient completed six questionnaires, leaving 12 out of 126 (9.5 %) questionnaires not filled out.

Overall, patients expressed satisfaction with their oART experience on Ethos. Nearly all patients, except one, found their time on the treatment couch acceptable. About 62 % of patients felt that their total time at the department was reasonable. More details on the questions can be found in Figs. A.4–7.

## 4 Discussion

This study evaluated the clinical implementation of an RTT-only CBCT-guided oART workflow by describing the training program, analyzing key workflow steps, and assessing patient experience. Establishing this workflow required a multidisciplinary effort, but with trained RTTs, support tools, and on-call ROs and/or MPEs, the RTT-only oART approach became feasible. The average session time was just 24-minutes, making it an efficient standard practice. RTTs play a crucial role in delivering high-quality, adaptive treatments, reducing clinician workload while improving efficiency, precision and patient care. Notably, patients reported positive experiences, reinforcing the importance of patient satisfaction in adopting innovative treatments.

A key distinction between MR-guided oART and CBCT-guided oART lies in the longer workflow duration and typically greater involvement of MPEs and ROs in MR-guided oART. Studies comparing the contour adaptation skills of RTTs and ROs in MR-guided oART have shown that while RTT-only workflows can reduce the need for RO and MPE support, this support is still more prominent than in CBCT-guided oART [32], [49]. However, in countries like the Netherlands and the UK [58], local regulations allow for RTT-only workflows, which leads to more efficiency. This indicates a growing need for international consensus regarding the responsibilities of RTTs in oART decision-making [59], [60].

In our department, we observed a 3.5-minute reduction in treatment time, a notable achievement, making RTT-only timeslots comparable to those needed for LoP [12]. This reduction is likely due to the extensive experience in our department with RTT-only LoP for bladder, cervix and rectal cancers [19], [29], [38], as well as online adaptive treatments with MR-guided oART [61]. The same RTT managing each oART step within a single session likely facilitated faster contour adjustments and plan evaluations, further enhancing overall workflow efficiency [25].

RTTs were trained to adapt the structures under time pressure, due to intrafraction bladder motion [37], [45], [58]. In over half the sessions, RTTs’ GTV delineations were similar to ROs’; in the other cases GTV was within acceptable PTV_boost_ margins (3 mm) and considered clinically acceptable. It is important to note that interobserver variation in bladder delineation by ROs can be up to 3 mm [62], [63]. Extensive training is crucial to reduce errors and minimize interobserver variability. AI-assisted tools may further improve consistency between RTTs and ROs and reducing interobserver variability in bladder delineation [32], [49]. Building experience shortened adjustment times and improved treatment efficacy, as observed in our department. MPEs or ROs were rarely needed during sessions, though they remained accessible via multidisciplinary agreements outlined in a traffic light protocol.

However, a limitation of our study was that the assessment of RTTs’ adjustments focused only on the GTV and excluded the lymph nodes, which were also a new addition to the RTT skill set. This decision was made due to the limited availability of the RO at that time. Although the nodes were not assessed in a separate way, the RTTs performed according to the multidisciplinary established agreements, and feedback on target coverage was provided through offline QA by the RO when necessary (and in real-time in the multidisciplinary workflow where ROs were present during all sessions). This feedback mechanism ensured that the accuracy of the RTTs’ delineations was continuously monitored and adjusted where needed. Despite this limitation, our findings suggest that properly trained RTTs can achieve a high level of efficiency and accuracy. For instance, RTTs frequently adjusted structures such as the bladder and GTV during the workflow, especially in response to interfraction bladder filling variations [37], [45], [58]. Another limitation was the lack of dosimetric impact analysis for the minor and major adjusted contours, which is addressed in a separate paper by Azzarouali et al. [25].

To ensure safety and treatment quality, online QA during treatment and offline QA posttreatment are essential [64], [65], [66]. ROs were consulted for OAR reviews, especially small bowel delineation, while MPEs addressed differences in MUs between plans. Offline QA feedback turned-out to be minimal, focusing on MUs differences, PTV margins, and OAR adjustments. As MR- and CBCT-guided oART are emerging techniques, collaborations are underway to establish QA guidelines [67], [68], with future studies evaluating the specific QA needs for this type of treatments [69].

Patient satisfaction remained high across various treatment types, including focal bladder cancer, palliative radiotherapy [57] and rectal cancer [38]. While patients appreciated the professionalism of the radiation support staff, there is room to improve the workflow duration and communication. Though health scores declined and side effects increased during oART − typical for radiotherapy – overall satisfaction with the process was positive. De Jong et al. reported similar findings, with an average session time of 34 min [38], while MRgRT session time averaged 45 min. Patient-reported outcomes (N = 89) indicating most patient tolerated MRgRT well, despite occasional discomfort from noise [61]. Similarly, in our study, all but one patient found the time on the couch acceptable, which was notably shorter than the time typically required for MRgRT.

Our department’s experience reflects the potential to reduce dependence on MPEs and ROs during sessions. Strict definitions of the responsibilities per profession, formalized by traffic light protocols, as well as availability of multidisciplinary support combined with online and offline QA [61], [62], [63] allow for the RTTs to perform many of the tasks traditionally handled by ROs and MPEs in such a way that quality of the treatment is ensured while the efficiency increases.

## 5 Conclusions

Developing and implementing RTT-only CBCT-guided oART is a multidisciplinary effort. With trained RTTs, support tools, and on-call ROs and MPEs, an autonomous RTT-only oART workflow for focal bladder cancer treatment is feasible and has received positive patient feedback. At our institute, we use this framework to implement RTT-only workflows for other indications. The RTT-only workflow for bladder cancer is now standard practice in our institute.

## Funding

This study was sponsored by a research grant from Varian a Siemens Healthineers Company.

## Declaration of competing interest

The authors declare the following financial interests/personal relationships which may be considered as potential competing interests: Amsterdam UMC is a member of the AIC: Adaptive Intelligence Consortium, which aims to coordinate international collaborative research relating to the Ethos Therapy™. Varian is a commercial member of the Ethos Consortium. Varian did not have any role in the study design, execution and reporting in this manuscript.

K. Goudschaal is sponsored by a research grant from Varian a Siemens Healthineers Company. S. Azzarouali received a grant from Varian a Siemens Healthineers Company. M. Hulshof was involved in several research collaborations with Varian a Siemens Healthineers Company, in the scope of the present work. A. Bel is involved in several research projects funded by Varian a Siemens Healthineers Company, in the scope of the present work. He is also involved in research projects supported by General Electric, Elekta, MED-LOGIX and Carl Reiner, outside de scope of this work. The other authors declare that they have no known competing financial interests or personal relationships that could have appeared to influence the work reported in this paper.

## References

[1] Fonteyne V., Ost P., Bellmunt J., Droz J.P., Mongiat-Artus P., Inman B. (2018). Curative treatment for muscle invasive bladder cancer in elderly patients: a systematic review. Eur Urol.

[2] Tan Z., Chen Z., Yao G., Mumin M.A., Wang Y., Zhu J. (2023). Neoadjuvant and adjuvant chemotherapy share equivalent efficacy in improving overall survival and cancer-specific survival among muscle invasive bladder cancer patients who undergo radical cystectomy: a retrospective cohort study based on SEER database. Transl Androl Urol.

[3] Garcia M.M., Gottschalk A.R., Brajtbord J., Konety B.R., Meng M.V., Roach M. (2014). Endoscopic gold fiducial marker placement into the bladder wall to optimize radiotherapy targeting for bladder-preserving management of muscle-invasive bladder cancer: feasibility and initial outcomes. PLoS One.

[4] Jiang D.M., Chung P., Kulkarni G.S., Sridhar S.S. (2020). Trimodality therapy for muscle-invasive bladder cancer: recent advances and unanswered questions. Curr Oncol Rep.

[5] Youssief Mohammed A.A., Gamal D.A., Abd Elzaher A.R., Hasaballah A. (2023). Trimodality therapy in which concurrent chemoradiation with concomitant boost in muscle invasive TCC urinary bladder cancer. Asian Pac J Cancer Prev.

[6] de Ruiter B.M., van Hattum J.W., Lipman D., de Reijke T.M., van Moorselaar R.J.A., van Gennep E.J. (2022). Phase 1 study of chemoradiotherapy combined with nivolumab ± ipilimumab for the curative treatment of muscle-invasive bladder cancer. Eur Urol.

[7] Milosevic M., Gospodarowicz M., Zietman A., Abbas F., Haustermans K., Moonen L. (2007). Radiotherapy for bladder cancer. Urology.

[8] Powles T., Bellmunt J., Comperat E., De Santis M., Huddart R., Loriot Y. (2022). ESMO Guidelines Committee. Electronic address: clinicalguidelines@esmo.org. Bladder cancer: ESMO Clinical Practice Guideline for diagnosis, treatment and follow-up. Ann Oncol.

[9] Oosterlinck W., Lobel B., Jakse G., Malmström P.U., Stöckle M., Sternberg C. (2002). European association of urology (EAU) working group on oncological urology. Guidelines on Bladder Cancer. *Eur Urol*.

[10] Brück K, Meijer RP, Boormans JL, Kiemeney LA, Witjes JA, van Hoogstraten LMC, et al; CRAC study group and the BlaZIB study group. Disease-free survival of patients with muscle-invasive bladder cancer treated with radical cystectomy versus bladder-preserving therapy: a nationwide study. *Int J Radiat Oncol Biol Phys*. 2023 Jul 28:S0360-3016(23)07686-1. doi: 10.1016/j.ijrobp.2023.07.027. 10.1016/j.ijrobp.2023.07.027 37517601

[11] Khalifa J, Roumiguié M, Pouessel D, Sargos P. Stratégie de préservation vésicale basée sur le traitement trimodal : quelle place dans la prise en charge du carcinome infiltrant de la vessie [Bladder-sparing trimodal therapy for muscle invasive bladder cancer]. *Cancer Radiother.* 2022 Oct;26(6-7):771-778. French. doi: 10.1016/j.canrad.2022.06.009. 10.1016/j.canrad.2022.06.009 35970682

[12] den Boer D., den Hartogh M.D., Kotte A.N.T.J., van der Voort van Zyp J.R.N., Noteboom J.L., Bol G.H. (2021). Comparison of Library of Plans with two daily adaptive strategies for whole bladder radiotherapy. Phys Imaging Radiat Oncol.

[13] Hijab A., Tocco B., Hanson I., Meijer H., Nyborg C.J., Bertelsen A.S. (2021). MR-guided adaptive radiotherapy for bladder cancer. Front Oncol.

[14] Lotz H.T., Pos F.J., Hulshof M.C., van Herk M., Lebesque J.V., Duppen J.C. (2006). Tumor motion and deformation during external radiotherapy of bladder cancer. Int J Radiat Oncol Biol Phys.

[15] Khouya A., Pöttgen C., Hoffmann C., Ringbaek T.P., Lübcke W. (2023). Adaptation time as a determinant of the dosimetric effectiveness of online adaptive radiotherapy for bladder cancer. Cancers (Basel).

[16] Shelley C, Hollingdale R, Bolt M, Rashid M, Reinlo S, et al. Radiographer-led, CBCT-guided online adaptive radiotherapy for muscle invasive bladder cancer using artificial intelligence. *Int J Rad Oncol*Biol Phys*. 114 (3), Supplement, 2022, Page e105, ISSN 0360-3016. doi:10.1016/j.ijrobp.2022.07.903.

[17] Huddart R., Hafeez S., Omar A., Alonzi R., Birtle A. (2023 Sep). Acute toxicity of hypofractionated and conventionally fractionated (chemo)radiotherapy regimens for bladder cancer: an exploratory analysis from the RAIDER Trial. Clin Oncol (r Coll Radiol).

[18] Hafeez S., Lewis R., Hall E., Huddart R., Raider, (2021 Jun). Trial Management Group. Advancing radiotherapy for bladder cancer: randomised phase II trial of adaptive image-guided standard or dose-escalated tumour boost radiotherapy (RAIDER). Clin Oncol (r Coll Radiol).

[19] Lutkenhaus L.J., Visser J., de Jong R., Hulshof M.C., Bel A. (2015). Evaluation of delivered dose for a clinical daily adaptive plan selection strategy for bladder cancer radiotherapy. Radiother Oncol.

[20] de Jong R., Lutkenhaus L., van Wieringen N., Visser J., Wiersma J., Crama K. (2016). Plan selection strategy for rectum cancer patients: An interobserver study to assess clinical feasibility. Radiother Oncol.

[21] Yan D., Lockman D., Brabbins D., Tyburski L., Martinez A. (2000). An off-line strategy for constructing a patient-specific planning target volume in adaptive treatment process for prostate cancer. Int J Radiat Oncol Biol Phys.

[22] Kong V.C., Taylor A., Rosewall T. (2017). Adaptive radiotherapy for bladder cancer-a systematic review. J Med Imaging Radiat Sci.

[23] Mitchell A., Ingle M., Smith G., Chick J., Diamantopoulos S., Goodwin E. (2022). Feasibility of tumour-focused adaptive radiotherapy for bladder cancer on the MR-linac. Clin Transl Radiat Oncol.

[24] Åström L.M., Behrens C.P., Calmels L., Sjöström D., Geertsen P., Mouritsen L.S. (2022). Online adaptive radiotherapy of urinary bladder cancer with full re-optimization to the anatomy of the day: Initial experience and dosimetric benefits. Radiother Oncol.

[25] Azzarouali S., Goudschaal K., Visser J., Hulshof M., Admiraal M., van Wieringen N. (2023). Online adaptive radiotherapy for bladder cancer using a simultaneous integrated boost and fiducial markers. Radiat Oncol.

[26] Lotz H.T., van Herk M., Betgen A., Pos F., Lebesque J.V., Remeijer P. (2005). Reproducibility of the bladder shape and bladder shape changes during filling. Med Phys.

[27] Lutkenhaus L.J., de Jong R., Geijsen E.D., Visser J., van Wieringen N., Bel A. (2016). Potential dosimetric benefit of an adaptive plan selection strategy for short-course radiotherapy in rectal cancer patients. Radiother Oncol.

[28] Horan N, Cooper JS. Radiation Cystitis and Hyperbaric Management. 2023 Jul 17. In: StatPearls [Internet]. Treasure Island (FL): StatPearls Publishing; 2023 Jan. Available from: https://www.ncbi.nlm.nih.gov/books/NBK470594. 29261976

[29] Collins S.D., Leech M.M. (2018). A review of plan library approaches in adaptive radiotherapy of bladder cancer. Acta Oncol.

[30] Kong V., Hansen V.N., Hafeez S. (2021). Image-guided adaptive radiotherapy for bladder cancer. Clin Oncol (r Coll Radiol).

[31] Krishnan A., Tsang Y.M., Stewart-Lord A. (2019). The impact of intra-fractional bladder filling on “Plan of the day” adaptive bladder radiotherapy. Tech Innov Patient Support Radiat Oncol.

[32] Rasing M.J.A., Sikkes G.G., Vissers N.G.P.M., Kotte A.N.T.J., Boudewijn J.H., Doornaert P.A.H. (2022). Online adaptive MR-guided radiotherapy: conformity of contour adaptation for prostate cancer, rectal cancer and lymph node oligometastases among radiation therapists and radiation oncologists. Tech Innov Patient Support Radiat Oncol.

[33] Intven M.P.W., de Mol van Otterloo S.R., Mook S., Doornaert P.A.H., de Groot-van Breugel E.N., Sikkes G.G. (2021). Online adaptive MR-guided radiotherapy for rectal cancer; feasibility of the workflow on a 1.5T MR-linac: clinical implementation and initial experience. Radiother Oncol.

[34] Bertholet J., Anastasi G., Noble D., Bel A., van Leeuwen R., Roggen T. (2020). Patterns of practice for adaptive and real-time radiation therapy (POP-ART RT) part II: Offline and online plan adaption for interfractional changes. Radiother Oncol.

[35] Botman R., Tetar S.U., Palacios M.A., Slotman B.J., Lagerwaard F.J., Bruynzeel A.M.E. (2019). The clinical introduction of MR-guided radiation therapy from a RTT perspective. Clin Radiat Oncol.

[36] McNair H.A., Wiseman T., Joyce E., Peet B., Huddart R.A. (2020). International survey; current practice in On-line adaptive radiotherapy (ART) delivered using Magnetic Resonance Image (MRI) guidance. Tech Innov Patient Support Radiat Oncol.

[37] Sibolt P., Andersson L.M., Calmels L., Sjöström D., Bjelkengren U., Geertsen P. (2020). Clinical implementation of artificial intelligence-driven cone-beam computed tomography-guided online adaptive radiotherapy in the pelvic region. Phys Imaging Radiat Oncol.

[38] de Jong R., Visser J., van Wieringen N., Wiersma J., Geijsen D., Bel A. (2021). Feasibility of Conebeam CT-based online adaptive radiotherapy for neoadjuvant treatment of rectal cancer. Radiat Oncol.

[39] Zwart L.G.M., Ong F., Ten Asbroek L.A., van Dieren E.B., Koch S.A., Bhawanie A. (2022). Conebeam computed tomography-guided online adaptive radiotherapy is feasible for prostate cancer patients. Phys Imaging Radiat Oncol.

[40] Åström L.M., Behrens C.P., Storm K.S., Sibolt P., Serup-Hansen E. (2022). Online adaptive radiotherapy of anal cancer: normal tissue sparing, target propagation methods, and first clinical experience. Radiother Oncol.

[41] Shelley C.E., Bolt M.A., Hollingdale R., Chadwick S.J., Barnard A.P., Rashid M. (2023). Implementing cone-beam computed tomography-guided online adaptive radiotherapy in cervical cancer. Clin Transl Radiat Oncol.

[42] Webster A., Hafeez S., Lewis R., Griffins C., Warren-Oseni K., Patel E. (2021). The development of therapeutic radiographers in imaging and adaptive radiotherapy through clinical trial quality assurance. Clin Oncol (r Coll Radiol).

[43] Stanley D.N., Harms J., Pogue J.A., Belliveau J.G., Marcrom S.R., McDonald A.M. (2023). A roadmap for implementation of kV-CBCT online adaptive radiation therapy and initial first year experiences. J Appl Clin Med Phys.

[44] Shepherd M., Graham S., Ward A., Zwart L., Cai B., Shelley C. (2021). Pathway for radiation therapists online advanced adapter training and credentialing. Tech Innov Patient Support Radiat Oncol.

[45] Koper E.J., Kamer M.J., de Jonge D.R., den Boer D., Hackett S., Onal C., Ozyar E. (2022). MR linac radiotherapy; a new personalized treatment approach.

[46] Hales R.B., Rodgers J., Whiteside L., McDaid L., Berresford J. (2020). Therapeutic radiographers at the helm: moving towards radiographer-led MR-guided radiotherapy. J Med Imaging Radiat Sci.

[47] Buijs M., Pos F., Frantzen-Steneker M., Rossi M., Remeijer P., Koetsveld F. (2021). Take Action Protocol: A radiation therapist led approach to act on anatomical changes seen on CBCT. Tech Innov Patient Support Radiat Oncol.

[48] van Beek S., Jonker M., Hamming-Vrieze O., Al-Mamgani A., Navran A., Remeijer P. (2019). Protocolised way to cope with anatomical changes in head & neck cancer during the course of radiotherapy. Tech Innov Patient Support Radiat Oncol.

[49] Willigenburg T., de Muinck K.DM., Peters M., Claes A., Lagendijk J.J.W., de Boer H.C.J. (2021). Evaluation of daily online contour adaptation by radiation therapists for prostate cancer treatment on an MRI-guided linear accelerator. Clin Transl Radiat Oncol.

[50] McNair H.A., Joyce E., O'Gara G., Jackson M., Peet B., Huddart R.A. (2021). Radiographer led online image guided adaptive radiotherapy: a qualitative investigation of the therapeutic radiographer role. Radiography (lond).

[51] Webster A., Francis M., Gribble H., Griffin C., Hafeez S. (2024). RAIDER Trial Management Group. Impact of on-trial IGRT quality assurance in an international adaptive radiotherapy trial for participants with bladder cancer. Radiother Oncol.

[52] Åström L.M., Sibolt P., Chamberlin H., Serup-Hansen E., Andersen C.E. (2024). Artificial intelligence-generated targets and inter-observer variation in online adaptive radiotherapy of bladder cancer. Phys Imaging Radiat Oncol.

[53] Chai X., van Herk M., van de Kamer J.B., Remeijer P., Bex A., Betgen A. (2010). Behavior of lipiodol markers during image guided radiotherapy of bladder cancer. Int J Radiat Oncol Biol Phys.

[54] de Ridder M., Gerbrandy L.C., de Reijke T.M., Hinnen K.A., Hulshof M.C.C.M. (2020). BioXmark® liquid fiducial markers for image-guided radiotherapy in muscle invasive bladder cancer: a safety and performance trial. Br J Radiol.

[55] Hulshof M.C., van Andel G., Bel A., Gangel P., van de Kamer J.B. (2007). Intravesical markers for delineation of target volume during external focal irradiation of bladder carcinomas. Radiother Oncol.

[56] Witjes J.A., Bruins H.M., Cathomas R., Compérat E.M., Cowan N.C., Gakis G. (2021). European association of urology guidelines on muscle-invasive and metastatic bladder cancer: summary of the 2020 guidelines. Eur Urol.

[57] Nelissen K.J., Versteijne E., Senan S., Rijksen B., Admiraal M., Visser J. (2023). Same-day adaptive palliative radiotherapy without prior CT simulation: Early outcomes in the FAST-METS study. Radiother Oncol.

[58] Betgen A., Bilderbeek J., Janssen T., Kaas J., Nowee M., Vijlbrief T. (2019). Real time IGART – changing responsibilities for RTTs on the MR-linac. Radiother Oncol.

[59] Dona Lemus O.M., Cao M., Cai B., Cummings M., Zheng D. (2024). Adaptive radiotherapy: next-generation radiotherapy. *Cancers* (basel).

[60] McNair H., Buijs M. (2019). Image guided radiotherapy moving towards real time adaptive radiotherapy; global positioning system for radiotherapy?. Tech Innov Patient Support Radiat Oncol.

[61] Tetar S.U., Bruynzeel A.M.E., Lagerwaard F.J., Slotman B.J., Bohoudi O., Palacios M.A. (2019). Clinical implementation of magnetic resonance imaging guided adaptive radiotherapy for localized prostate cancer. Phys Imaging Radiat Oncol.

[62] Meijer G.J., Rasch C., Remeijer P., Lebesque J.V. (2003). Three-dimensional analysis of delineation errors, setup errors, and organ motion during radiotherapy of bladder cancer. Int J Radiat Oncol Biol Phys.

[63] Nishioka K., Shimizu S., Kinoshita R., Inoue T., Onodera S., Yasuda K. (2013). Evaluation of inter-observer variability of bladder boundary delineation on cone-beam CT. Radiat Oncol.

[64] Huq M.S., Fraass B.A., Dunscombe P.B., Gibbons J.P., Ibbott G.S., Mundt A.J. (2016). The report of Task Group 100 of the AAPM: application of risk analysis methods to radiation therapy quality management. Med Phys.

[65] Glide-Hurst C.K., Lee P., Yock A.D., Olsen J.R., Cao M., Siddiqui F. (2021). Adaptive radiation therapy (ART) strategies and technical considerations: a state of the ART review from NRG oncology. Int J Radiat Oncol Biol Phys.

[66] Okamoto H., Igaki H., Chiba T., Shibuya K., Sakasai T., Jingu K. (2022). Practical guidelines of online MR-guided adaptive radiotherapy. J Radiat Res.

[67] Hurkmans C, Bibault J-E, Brock KK, van Elmpt W, Feng M, Fuller CD, et al. A joint ESTRO and AAPM guideline for development, clinical validation and reporting of artificial intelligence models in radiation therapy, *Radiother Oncol,* 197, 2024, 110345, ISSN 0167-8140. doi: 10.1016/j.radonc.2024.110345. 10.1016/j.radonc.2024.110345 38838989

[68] Hackett S. and Klüter S. Quality assurance for online adaptive radiotherapy. [Online]. Available: https://www.estro.org/Workshops/2023-Physics-Workshop-Science-in-Development/Quality-assurance-for-online-adaptive-radiotherapy.

[69] Zhao X., Stanley D.N., Cardenas C.E., Harms J., Popple R.A. (2023). Do we need patient-specific QA for adaptively generated plans? retrospective evaluation of delivered online adaptive treatment plans on Varian Ethos. J Appl Clin Med Phys.

