# Supplementary material for: Clinical implementation of RTT-only CBCT-guided online adaptive focal radiotherapy for bladder cancer
Source: Clin Transl Radiat Oncol. 2024 Nov 2;50:100884. doi: 10.1016/j.ctro.2024.100884 (PMC11570400; doi:10.1016/j.ctro.2024.100884)
Supplement: Supplementary Data 1 [file mmc1.docx]

**Appendix A. Supplementary data**

**Supplementary data 1: Patient characteristics, pretreatment information, and details from our previous study by Azzarouali et al.**

Table A.1: Patient characteristics including sex, age, tumor stage and TP technique.

| **Multidisciplinary workflow** | | | |  | **RTT-only workflow** | | | |
| --- | --- | --- | --- | --- | --- | --- | --- | --- |
| **Patient** | **Sex** | **Age** | **Tumor stage** according to the 2009 TNM classification [56], [70], [71] |  | **Patient** | **Sex** | **Age** | **Tumor stage** according to the 2009 TNM classification [56], [70], [71] |
| 1 | M | 64 | (cT2N0M0)G3 |  | 1 | M | 75 | (cT2N0M0)G3 |
| 2 | M | 86 | (cT2N0M0)G3 |  | 2 | M | 81 | (cT2-3N0M0)G3 |
| 3 | M | 79 | (cT2N0M0)G3 |  | 3* | F | 61 | (cT2N0M0)G3 |
| 4* | M | 87 | (cT2N0M0)G3 |  | 4* | M | 89 | (cT3N0M0)G3 |
| 5* | M | 63 | (cT2N0M0)G3 |  | 5* | F | 82 | (cT3N0M0)G3 |
| 6 | F | 67 | (cT2N0M0)G3 |  | 6* | M | 70 | (cT2N0M0)G3 |
| 7 | F | 71 | (cT2N0M0)G3 |  | 7 | M | 87 | (cT2N0M0)G3 |
| 8 | F | 78 | (cT2N0M0)G3 |  | 8* | M | 83 | (cT2N0M0)G3 |
| 9* | F | 66 | (cT2N0M0)G3 |  | 9* | M | 55 | (cT2N0M0)G3 |
| 10 | M | 65 | (cT2N0M0)G3 |  | 10* | M | 64 | (cT3N0M0)G3 |
| 11 | M | 68 | (cT2N0M0)G3 |  | 11* | M | 58 | (cT2N0M0)G3 |
| 12 | M | 59 | (cT2N0M0)G3 |  | 12 | M | 56 | (cT3N0M0)G3 |
| 13 | M | 72 | (cT2N0M0)G3 |  | 13 | F | 78 | (cT2N0M0)G3 |
| 14 | M | 79 | (cT2N0M0)G3 |  | 14 | M | 67 | (cT2N1M0)G3 |
| Median age 72 (range, 59-87 years).  TP technique:  Simultaneous integrated boost (SIB) in white  Sequential boost in white with a * | | | |  | Median age 72 (range, 55-89 years).  TP technique:  Simultaneous integrated boost (SIB) in white  Sequential boost in white with a * | | | |

**Pretreatment information**

Table A.2: Information on SIB and sequential boost treatment.

|  | **SIB** | **Sequential boost** |
| --- | --- | --- |
| **Drinking instructions** | Full Bladder preparation: patients were asked to drink 0.3 L of water after voiding the bladder and subsequently refrain from drinking 1.5 hours prior to CT/oART. | Full Bladder preparation: patients were asked to drink 0.3 L of water after voiding the bladder and subsequently refrain from drinking 1.5 hours prior to CT/oART. |
|  |  | Empty bladder preparation: patients were asked to void the bladder 1.5 hours prior to CT/oART and subsequently refrain from drinking. |
| **Number of pretreatment scans** | Two pCT were made.  The first pCT was made with a full bladder to delineate GTV, CTV and OAR, including the small bowel if the GTV was in the cranial part of the bladder.  The second pCT was made 15 minutes after the first, to generate patient-specific margins if intrafraction bladder filling needed them. | Two pCT were made.  The first pCT was made with a full bladder to delineate GTV and OAR, including small bowel if the GTV was in the cranial part of the bladder.  The second pCT was made to generate CTVelective and OAR with an empty bladder. |
| **Margins** | GTV-CTV and CTV-PTV margin of 5mm were used.  Urethra and pelvic lymph nodes CTV to PTV margin of 5-7mm. | GTVp-CTVp margin of 3mm and CTV-PTV margin of 5mm were used.  Urethra and pelvic lymph nodes CTV to PTV margin of 5 (nodes) 7mm (bladder). |
| **TPS** | Ethos, version 1.1  Prioritized clinical goals for optimization of the final dose distribution  VMAT, 3 arcs, 6 MV FFF | Ethos, version 1.1  Prioritized clinical goals for optimization of the final dose distribution  IMRT, 9 fields, 6MV FFF |

**Details from our previous study by Azzarouali et al. on bladder cancer oART using SIB and fiducial markers [25]**

**Objectives:**

1. Assess the quality of online treatment plans.
2. Evaluate the quality of automatic target delineation.
3. Measure the duration of main workflow steps and total on-couch time.
4. Examine performance in the presence of fiducial markers for tumor bed localization.

**Study Details:**

- **Number of Patients:** 15 patients treated with Simultaneous Integrated Boost (SIB).

**Results:**

1. **Quality of Treatment Plans:**
   - **Target Coverage:**
     - Evaluation of target coverage and V95% outside the target for both scheduled and adaptive plans.
     - All 300 adaptive plans met clinical requirements.
     - Less tissue outside the target received 55 Gy.
2. **Target Delineation:**
   - **Manual Adjustments:**
     - Manual adjustments were needed in 67% of sessions.
     - 96% of GTV (Gross Tumor Volume) enlargements were observed, resulting in a 1% improvement in target coverage.
3. **Workflow Duration:**
   - **On-Couch Time:**
     - Median on-couch time: 22 minutes.
     - One-third of session time was spent reoptimizing the treatment plan.
   - **Optimization:**
     - Improved automatic delineation and faster reoptimization would shorten session times.
4. **Influence of Fiducial Markers:**
   - Fiducial markers were visible on CBCTs, facilitating consistent daily tumor localization.

**Conclusion:** AI-driven CBCT-guided oART, aided by fiducial markers, is feasible for bladder cancer, including with SIB.

**Average session time per patient: SIB versus sequential boost**

- - **Multidisciplinary workflow**: 11 SIB sessions (average session time of 28.6 minutes per patient) versus 3 sequential boost sessions (average session time of 24 minutes per patient).

Total average session time: 27.6 minutes (see Fig. 2).

- - **RTT-only workflow**: 6 SIB sessions (average session time of 28 minutes per patient) versus 8 sequential boost sessions (average session time of 21.1 minutes per patient).

Total average session time: 24.1 minutes (see Fig. 2).

**Supplementary data 2: Additional training scheme table**

Table A.3: Table visualizing the training of the RTT, RO and MPE involved in oART. The training program includes general oART workflow training, followed by target volume training specific to each target area. Abbreviations: h (hour), min (minutes), prep (preparing).

| **Additional training for each RTT/RO/MPE involved in oART**  **General** | **RTT** | **RO** | **MPE** |
| --- | --- | --- | --- |
| Bladder target volume training session by the RO | 1 h | 1 h (+ prep time) |  |
| Treatment planning by the MPE specialized on oART TP | 1 h | 1 h | 1 h (+ prep time) |
| Practice GTV/CTV/OAR delineation in-house training | 2 h |  |  |
| Perform oART in the Emulator | 20 h | 1-2 h with a specialist RTT | 1-2 h |
| Examination of the oART workflow in the Emulator | 1-2 h with a specialist RTT |  |  |
| RTT performed 10 sessions of clinical training under supervision of a trained RTT | 5 h |  |  |
| Multidisciplinary briefing before oART | 10 min | 10 min | 10 min |
| Case meetings held every two months | 1 h (+ prep time) | 1 h | 1 h |

**Supplementary data 3: Traffic light protocol**

**
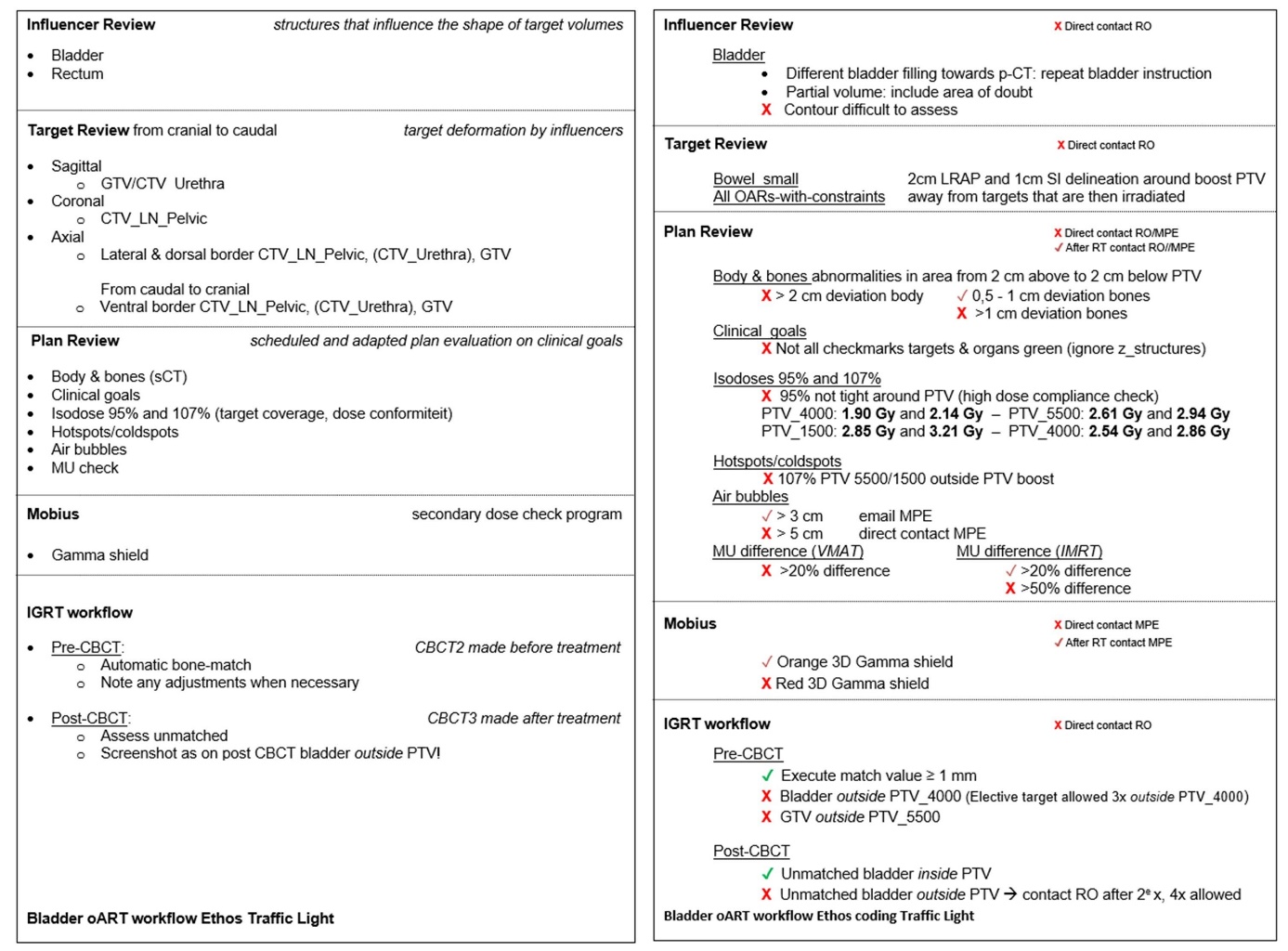
**Figure A.1**:** Traffic light protocol used during bladder oART in the RTT-only workflow. Main oART workflow steps (left) and coding of the main steps (right). A green light is given when all conditions are fulfilled and the treatment can continue. If an intervention by MPE or RO is required after a radiation session due to predefined minor changes, the light is orange. For predefined major changes, the light turns red, implying that the RO and/or MPE had to be consulted immediately before treatment could be started. RTTs were trained to adjust organs at risk (OAR) (sigmoid) only when in/close to the targets that are irradiated. The small bowel was only delineated and adjusted within a 2cm ring to the PTV of the boost (PTV_boost_) for lowering the maximum dose.

Condition and required action [25], [38]

Image Quality image quality of CBCT1 needs to be good enough to proceed with oART

Influencer Review structures that influence the shape of target volumes on CBCT1

Target Review target deformation by influencers

Plan Review reference/pre-treatment and adapted plan evaluation on clinical goals

Mobius secondary dose check program

IGRT workflow Pre-oART CBCT made before treatment to check patient position

Post-oART CBCT made after treatment to check intrafraction variation

LRAP Left Right Anterior Posterior

SI Superior Inferior

**Supplementary data 4: Multidisciplinary contact**


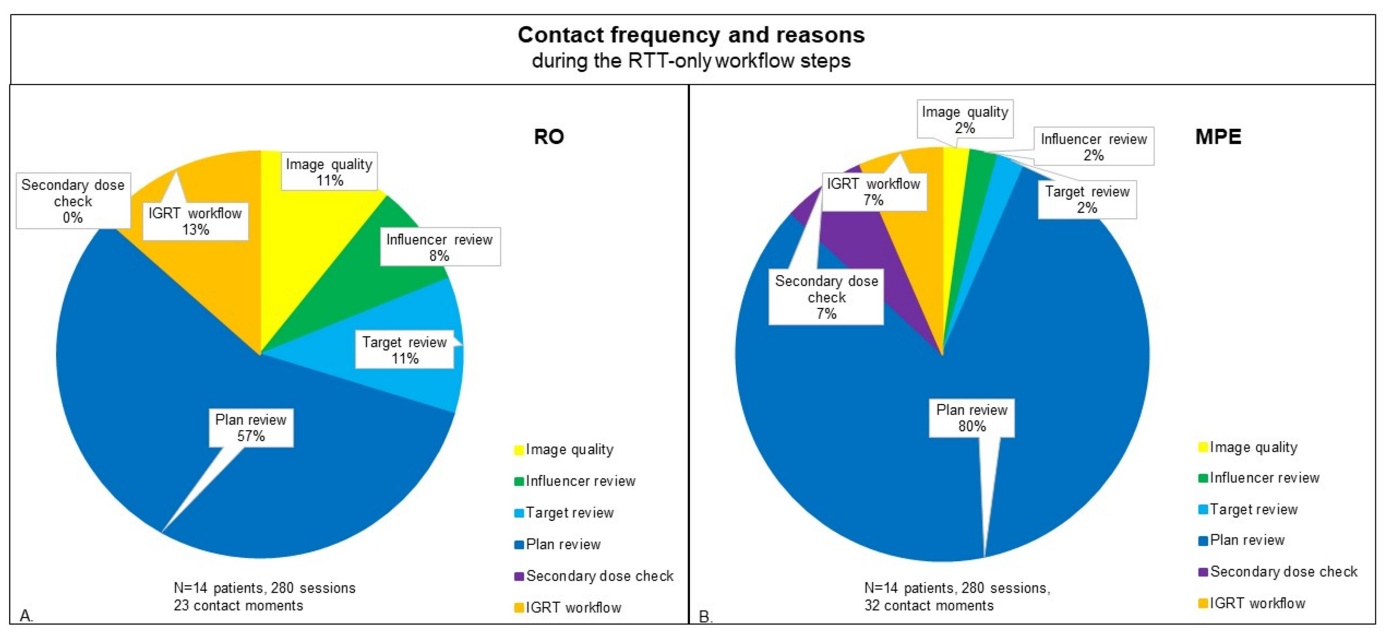
Figure A.2: Contact frequency and reasons during the RTT-only workflow steps. A: contact with RO (23 times) and B: contact with MPE (32 times).The IGRT workflow takes place after the online adaptive plan was made and consists of pre-oART CBCT (CBCT2), the radiotherapy treatment itself and the post-oART CBCT (CBCT3).

Table A.4: Example of questions that need to be answered in different RTT-only workflow steps.

| **Different RTT-only workflow steps** | **Following questions need to be answered** |
| --- | --- |
| Image quality | Is the image quality of CBCT1 good enough to proceed?  Is the patient positioning and target area stable enough?  Is there air in the bowel? |
| Influencer review | Is the bladder visible?  Do the bladder and rectum need adjustments? |
| Target review | Are the targets (de)formed correctly?  Does an OAR (such as small bowel) need adjustments? |
| Plan review | Are all clinical goals checked?  Is the dosimetry according to predefined protocol?  Is the amount of MUs within predefined threshold value? |
| Secondary dose check | Are there more or less MUs? |
| IGRT workflow | Did the target move before oART?  Did the target move after oART? |

**Supplementary data 5: Qualitative assessment of the GTV delineation on CBCT1 within the RTT-only workflow by the RO, retrospectively**

**
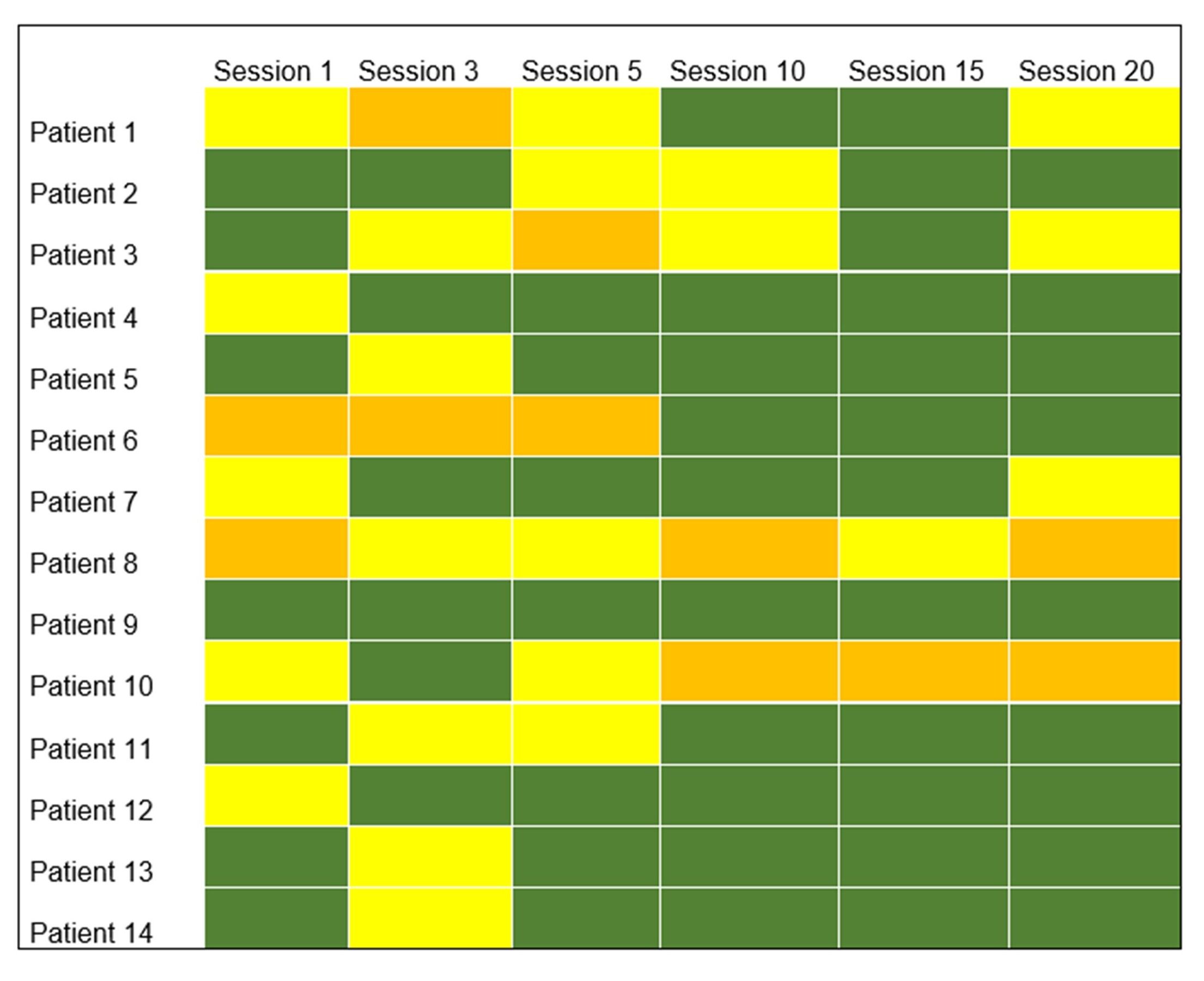
**Figure A.3: Qualitative assessment of the GTV delineation displayed in a color distribution per patient, N=14 patients, total 86 sessions. No edit required (green), clinical acceptable (yellow), major adjustments required (orange). Unacceptable delineation did not occur. See Figure 5 in the main article for an overview of this data.

**Supplementary data 6: Questionnaires**

In-house developed satisfaction questionnaire (translated from Dutch below)

The questionnaire is based on a previously designed in-house PRO-Q questionnaire to evaluate patient tolerance [72].

Twenty-one patients, instead of twenty-eight, completed the questionnaires. This was because the multidisciplinary workflow had already begun with the first four patients before the questionnaires were approved by the METC, and three patients dropped out due to changes in their health.

All questions had the following answer options: 1 – not, 2 – little, 3- moderate, 4 – a lot. Negative outcomes or dissatisfaction was defined by scoring 4 for questions 1-3, 5-7, 10; and for questions 4, 8, and 9 by scoring 1.

Given the intensity of treatment on this radiation device, we would like to evaluate your experience. Could you please indicate how much each of these questions applies to your experience?

1. Was the explanation of the treatment preparation and procedure understandable?
2. Did the radiation oncologist adequately inform you regarding possible side effects?
3. Were you satisfied with the length of time between your consultation with the radiation oncologist and the commencement of your treatment?
4. Did you experience anxiety during your time on the treatment couch?
5. Was the amount of time spent on the treatment couch acceptable?
6. Was communication during treatment understandable?
7. Did you think the radiation device's support staff was professional in their approach?
8. Did symptoms from your disease increase during the treatment?
9. Did you find the total time spent at the department to be long?
10. In case you are referred for radiotherapy again, would you prefer the same treatment pathway?

Original satisfaction questionnaire in Dutch (Version 2, June 16, 2022) [57]

Vanwege de intensieve behandeling op dit bestralingsapparaat, willen wij graag evalueren hoe u dit hebt ervaren. Wilt u aangeven in welke mate onderstaande vragen op u van toepassing zijn?

Opties: 1 – helemaal niet, 2- een beetje, 3 – nogal, 4 - heel erg

1. Was de uitleg over de procedure van de voorbereiding en de behandeling begrijpelijk?
2. Was u door de bestralingsarts goed voorbereid op de te verwachten bijwerkingen?
3. Was de wachttijd tussen het eerste consult bij de bestralingsarts en start van de behandeling acceptabel?
4. Voelde u zich angstig tijdens het liggen in het bestralingstoestel?
5. Was de tijd dat u op het bestralingstoestel lag acceptabel?
6. Was de communicatie tijdens de behandeling begrijpelijk?
7. Heeft u de begeleiding van het personeel op het bestralingstoestel als professioneel ervaren?
8. Zijn de klachten die u als gevolg van uw ziekte heeft, toegenomen tijdens de behandeling?
9. Heeft u de totale duur dat u op de afdeling aanwezig was als lang ervaren?
10. Indien u opnieuw verwezen wordt voor een bestraling, vind u het dan prettig om op dezelfde manier behandeld te worden?

In-house developed Baseline questionnaire (translated from Dutch below)

All questions had the following answer options:

Options question 1: 1 – not, 2 – seldom, 3 – little, 4 – moderate, 5 – a lot

Options question 2: 1 – poor, 2 – moderate, 3 – good, 4 – very good, 5 – excellent

Negative outcomes or dissatisfaction was defined by scoring 5 for questions 1; and for question 2 by scoring 1.

We want to assess how you felt about the treatment because of how intense it was on this radiation device. Could you say to what extent these questions apply to you? This first questionnaire is a zero measurement.

1. Do you suffer from the following complains:
   1. Intestinal complaints
   2. Difficulty holding stools
   3. Difficulty holding up pee
   4. Difficulty urinating completely
   5. Bladder spasms
   6. Pain when urinating
2. How would you rate your overall health at this moment?

Original Baseline questionnaire in Dutch (Version 2, June 14, 2022)

Opties vraag 1: 1 – nooit, 2 – zelden, 3 – soms, 4 – vaak, 5 – altijd

Opties vraag 2: 1 – slecht, 2 – matig, 3 – goed, 4 – zeer goed, 5 – uitstekend

Deze eerste vragenlijst is een nulmeting.

1. Heeft u last van de volgende klachten
   1. Darmklachten
   2. Moeite met ontlasting ophouden
   3. Moeite met plas ophouden
   4. Moeite met volledig uit plassen
   5. Blaaskrampen
   6. Pijn bij het plassen
2. Hoe zou u op dit moment uw algehele gezondheid noemen?

In-house developed Follow-up questionnaire (translated from Dutch below)

All questions had the following answer options:

Options question 1 - 4: 1 – not, 2 – seldom, 3 – little, 4 – moderate, 5 – a lot

Options question 5: 1 – poor, 2 – moderate, 3 – good, 4 – very good, 5 – excellent

Negative outcomes or dissatisfaction was defined by scoring 5 for questions 1; and for question 2-4 by scoring 1 and for questions 5 by scoring 1.

We want to assess how you felt about the treatment because of how intense it was on this radiation device. Could you say to what extent these questions apply to you? This questionnaire looks back at the past five sessions

1. Do you suffer from the following complains:

- 1. Intestinal complaints
  2. Difficulty holding stools
  3. Difficulty holding up pee
  4. Difficulty urinating completely
  5. Bladder spasms
  6. Pain when urinating

2. I have been able to lie still during the past five radiation treatments. (Irradiation is the total duration time of lying on the treatment couch).

3. The bladder filling instruction was easy to maintain during the past five radiation treatments.

4. I was able to keep my arms in the same position during the past five radiation treatments.

5. How would you rate your overall health during the past five radiation treatments?

Original Follow-up questionnaire in Dutch (Version 2, June 14, 2022)

Opties vraag 1: 1 – nooit, 2 – zelden, 3 – soms, 4 – vaak, 5 – altijd

Opties vraag 5: 1 – slecht, 2 – matig, 3 – goed, 4 – zeer goed, 5 – uitstekend

1.Heeft u last van de volgende klachten

- 1. Darmklachten
  2. Moeite met ontlasting ophouden
  3. Moeite met plas ophouden
  4. Moeite met volledig uit plassen
  5. Blaaskrampen
  6. Pijn bij het plassen

2.Ik ben goed in staat geweest om tijdens de afgelopen vijf bestralingen stil te blijven liggen. (Bestraling is de totale duur van het op tafel liggen)

3. De instructie met betrekking tot de blaasvulling was makkelijk vol te houden tijdens de afgelopen vijf bestralingen.

4. ik was goed in staat om mijn armen tijdens de afgelopen vijf bestralingen in dezelfde positie te houden.

5.Hoe zou u tijdens de afgelopen vijf bestralingen uw algehele gezondheid noemen?


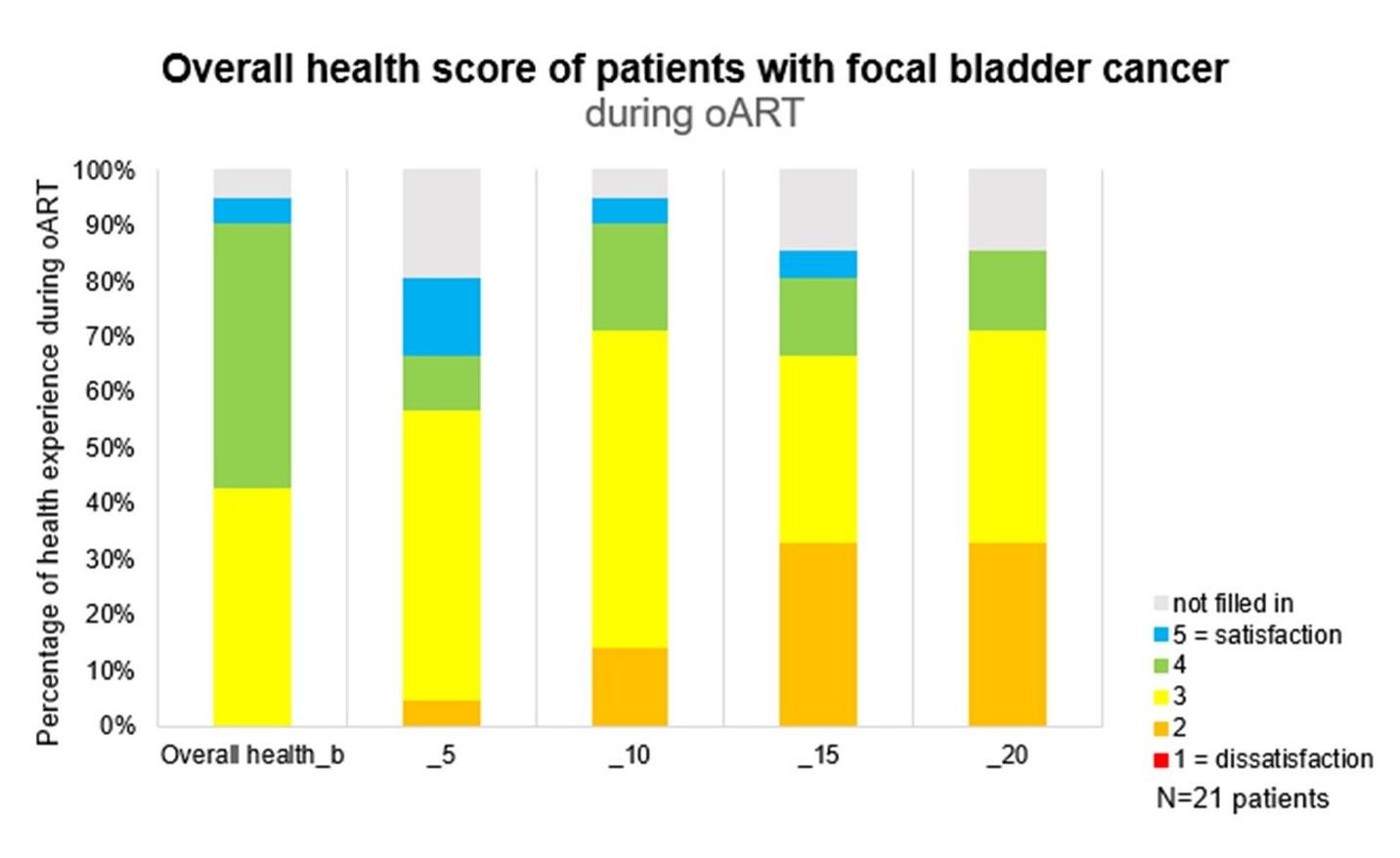


Figure A.4: Stacked column chart visualizing trends in overall health score from baseline (_b) through oART sessions 5, 10, 15 and 20. 5 point Likert scale score from 1 (dissatisfaction) up to 5 (satisfaction). 1=poor; 2=moderate, 3=good, 4=very good, 5=excellent.


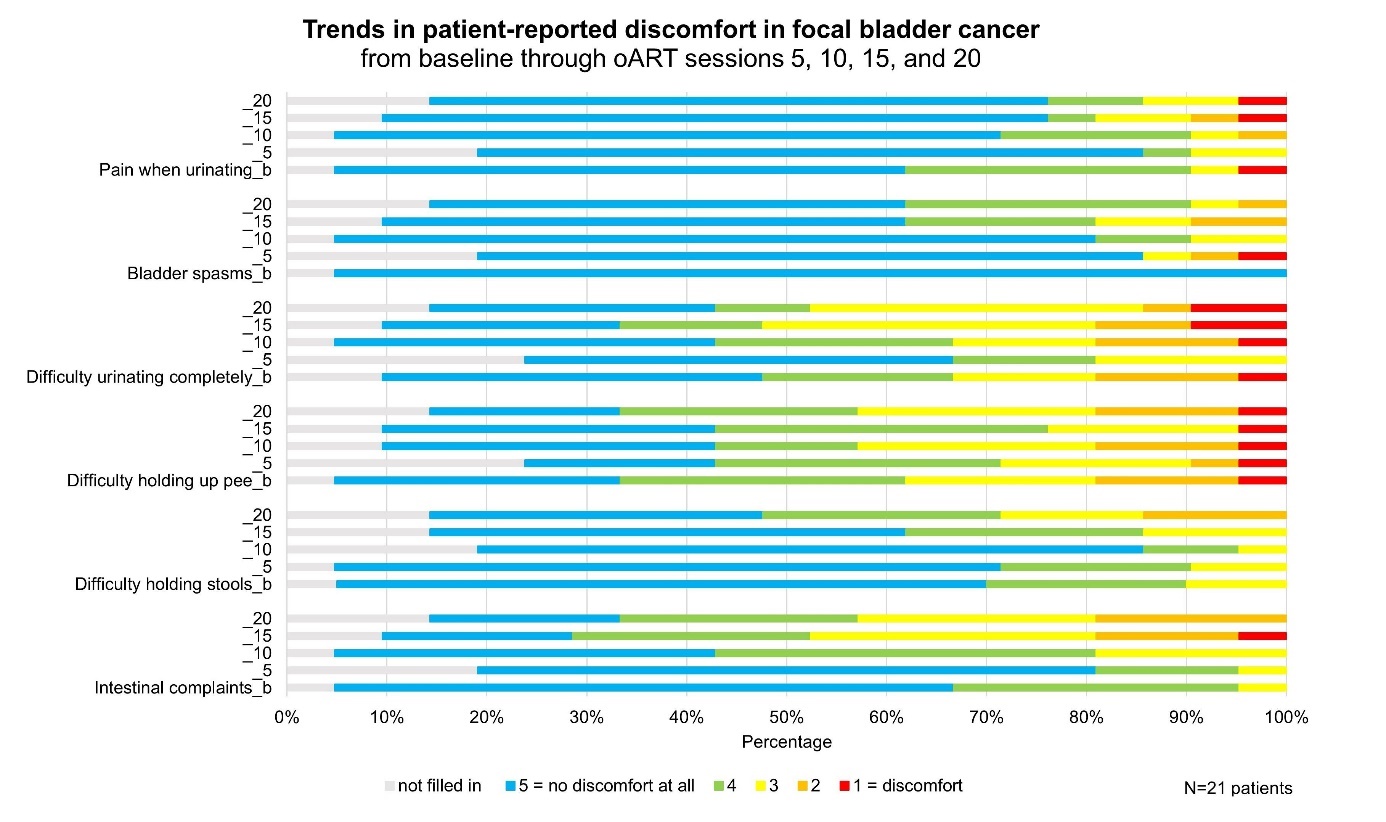
Figure A.5: Stacked column chart visualizing trends in patient-reported discomfort in focal bladder cancer from baseline (_b) through oART sessions 5, 10, 15 and 20. 5 point Likert scale score from 1 (discomfort) up to 5 (no discomfort at all). 1=a lot; 2=moderate, 3=little, 4=seldom, 5=not.


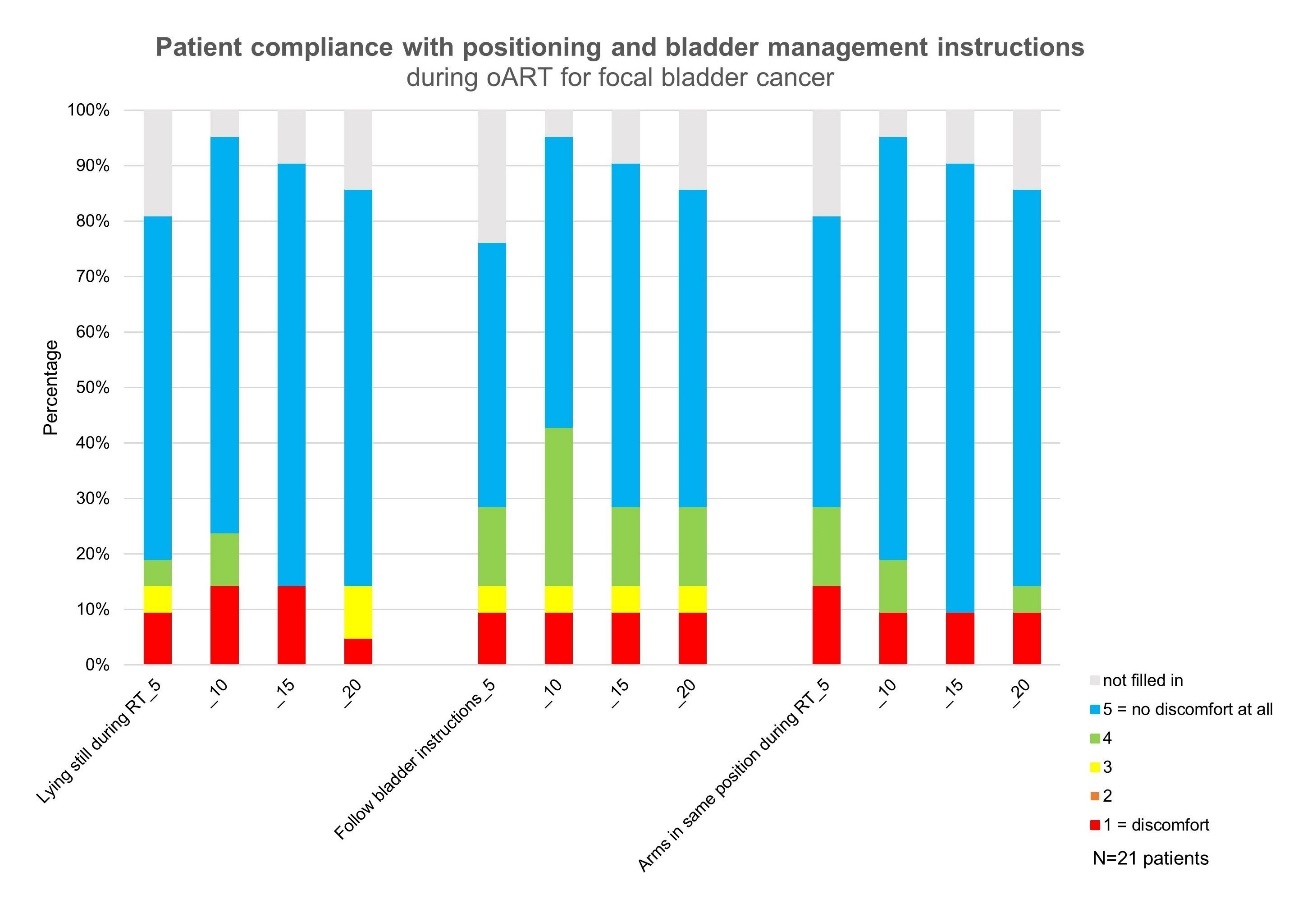
Figure A.6: Stacked column chart from session 5 (_5) to session 20 (_20), visualizing trends in patient compliance with positioning and bladder management instructions during oART for focal bladder cancer. 5 point Likert scale score from 1 (discomfort) up to 5 (no discomfort at all). 1=a lot; 2=moderate, 3=little, 4=seldom, 5=not.


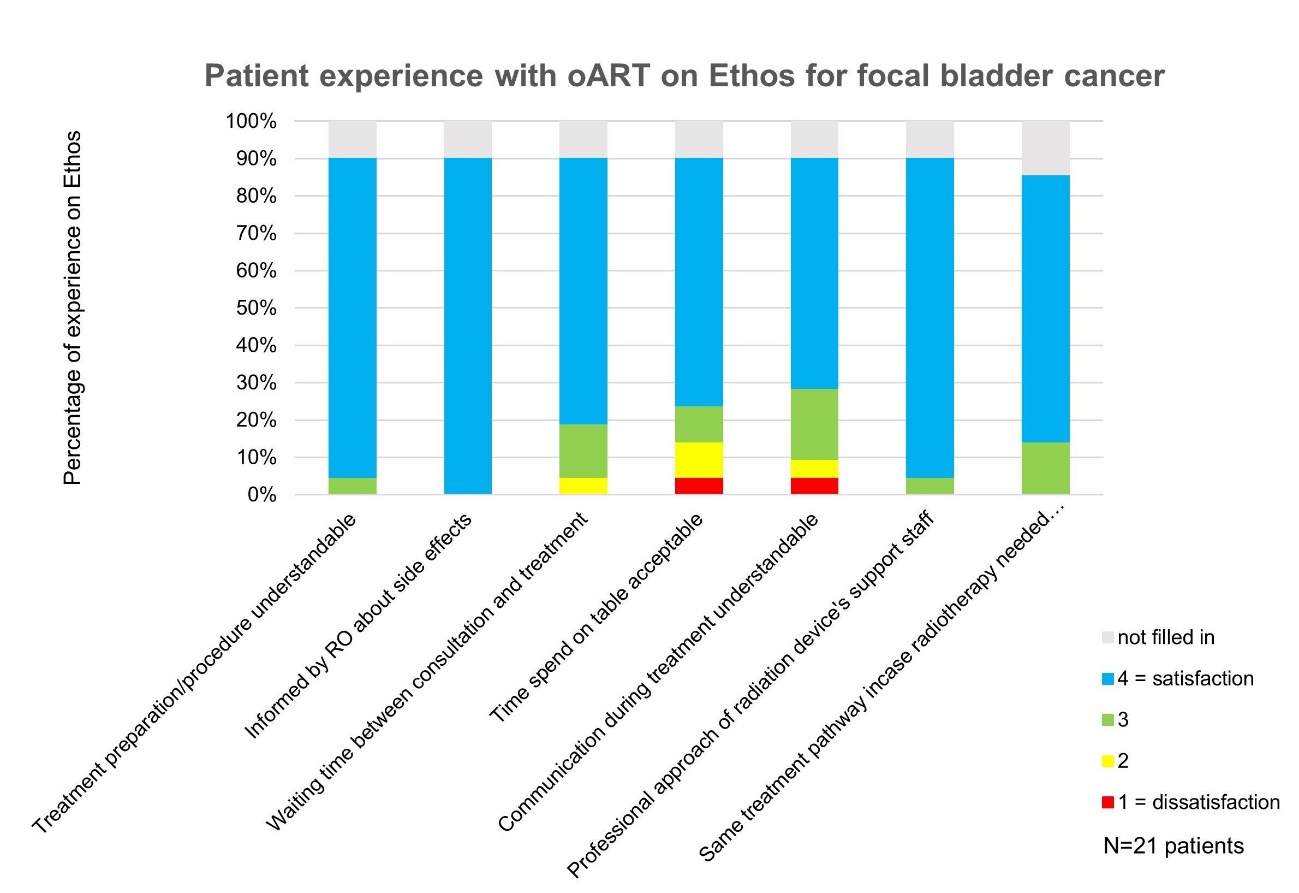
Figure A.7a: Stacked column chart visualizing patient experience with oART on Ethos for focal bladder cancer. 4 point Likert scale score from 1 (dissatisfaction) up to 4 (satisfaction). 1= not; 2=little, 3=moderate, 4=a lot.


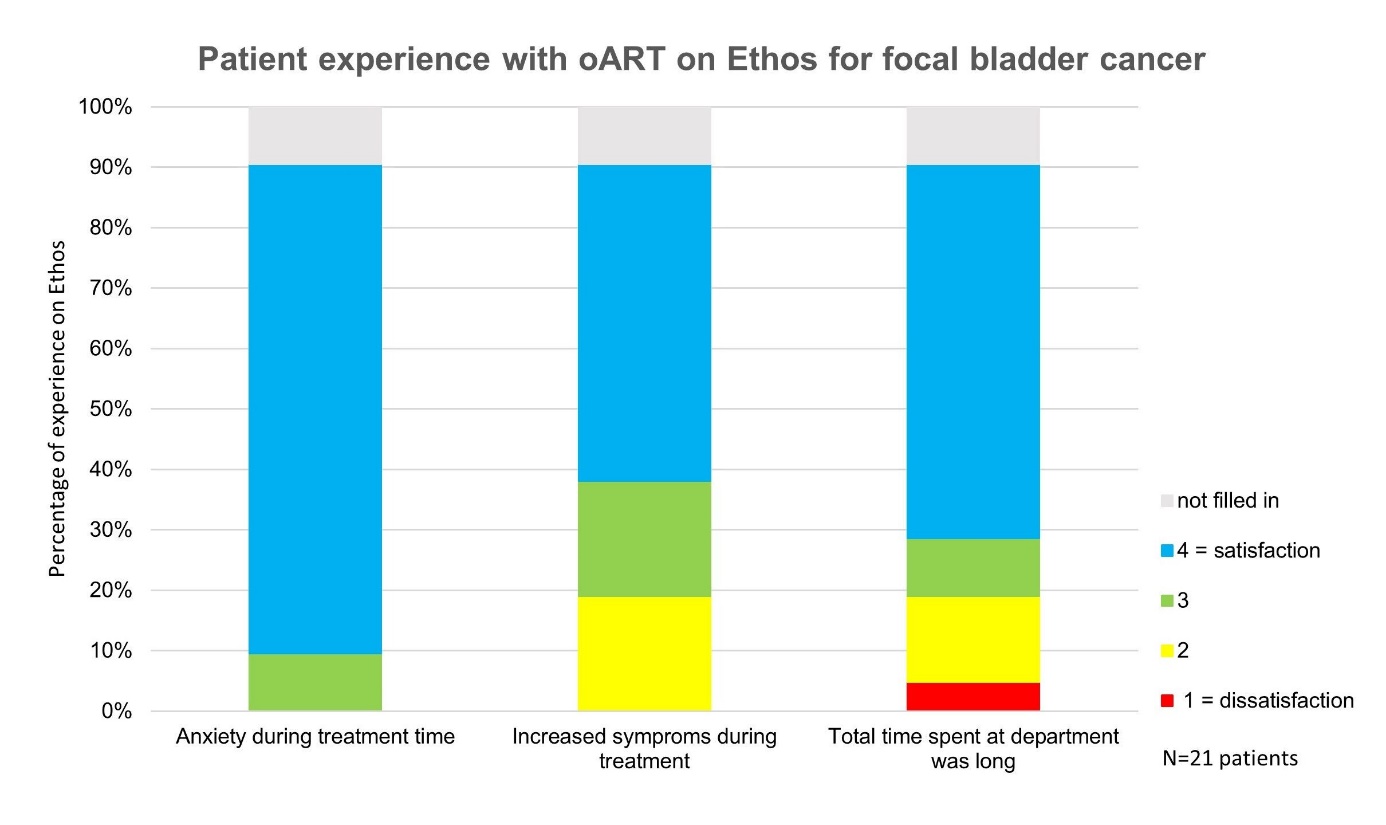
Figure A.7b: Stacked column chart visualizing patient experience with oART on Ethos for focal bladder cancer. 4 point Likert scale score from 1 (dissatisfaction) up to 4 (satisfaction). 1= a lot; 2=moderate, 3=little, 4=not.

**References**

These references are the ones used in the Supplementary data. A complete list of all references can be found in the manuscript.

[25] Azzarouali S., Goudschaal K., Visser J., Hulshof M., Admiraal M., van Wieringen N., et al. Online adaptive radiotherapy for bladder cancer using a simultaneous integrated boost and fiducial markers. *Radiat Oncol*. 2023 Oct 6;18(1):165. doi: 10.1186/s13014-023-02348-8.

[38] de Jong R., Visser J., van Wieringen N., Wiersma J., Geijsen D., Bel A. Feasibility of Conebeam CT-based online adaptive radiotherapy for neoadjuvant treatment of rectal cancer. *Radiat Oncol.* 2021 Jul 23;16(1):136. doi: 10.1186/s13014-021-01866-7.

[56] Witjes JA., Bruins HM., Cathomas R., Compérat EM., Cowan NC., Gakis G., et al. European Association of Urology Guidelines on Muscle-invasive and Metastatic Bladder Cancer: Summary of the 2020 Guidelines. *Eur Urol.* 2021 Jan;79(1):82-104. doi: 10.1016/j.eururo.2020.03.055.

[57] Nelissen KJ., Versteijne E., Senan S., Rijksen B., Admiraal M., Visser J., et al. Same-day adaptive palliative radiotherapy without prior CT simulation: Early outcomes in the FAST-METS study. *Radiother Oncol.* 2023 May;182:109538. doi: 10.1016/j.radonc.2023.109538.

[70] Witjes JA., van der Heijden AG., Nooter RI., van Rhijn BWG., Smilde TJ., Bergman AM., et al. Richtlijn Blaascarcinoom - Nederlandse samenvatting van de EAU guidelines on bladder cancer. Pp. 11-14, 2016.

[71] Sobin LH., Gospodarowicz MK., Wittekind C. TNM Classification of Malignant Tumours, 8^th^ Edition. Wiley-Blackwell 2016;7:262-265. ISBN:978-1-119-26357-9.

[72] Tetar S., Bruynzeel A., Bakker R., Jeulink M., Slotman BJ., et al. Patient-reported Outcome Measurements on the Tolerance of Magnetic Resonance Imaging-guided Radiation Therapy. *Cureus.* 2018 Feb 27;10(2):e2236. doi: 10.7759/cureus.2236.
